# Customized tracheal design using 3D printing of a polymer hydrogel: influence of UV laser cross-linking on mechanical properties

**DOI:** 10.1186/s41205-019-0049-8

**Published:** 2019-08-02

**Authors:** Ana Filipa Cristovão, David Sousa, Filipe Silvestre, Inês Ropio, Ana Gaspar, Célia Henriques, Alexandre Velhinho, Ana Catarina Baptista, Miguel Faustino, Isabel Ferreira

**Affiliations:** 1 i3N/CENIMAT, Department of Materials Science, Lisbon, Portugal; 2 FAB-LAB, Lisbon, Portugal; 3 i3N/CENIMAT, Department of Physics, Faculty of Science and Technology, Universidade NOVA de Lisboa, Campus de Caparica, 2829-516 Caparica, Portugal

**Keywords:** 3D printing, Biopolymer, In-situ UV laser polymerization, Mechanical properties, Tracheal 3D model

## Abstract

**Background:**

The use of 3D printing of hydrogels as a cell support in bio-printing of cartilage, organs and tissue has attracted much research interest. For cartilage applications, hydrogels as soft materials must show some degree of rigidity, which can be achieved by photo- or chemical polymerization. In this work, we combined chemical and UV laser polymeric cross-linkage to control the mechanical properties of 3D printed hydrogel blends. Since there are few studies on UV laser cross-linking combined with 3D printing of hydrogels, the work here reported offered many challenges.

**Methods:**

Polyethylene glycol diacrylate (PEGDA), sodium alginate (SA) and calcium sulphate (CaSO_4_) polymer paste containing riboflavin (vitamin B2) and triethanolamine (TEOHA) as a biocompatible photoinitiator was printed in an extrusion 3D plotter using a coupled UV laser. The influence of the laser power on the mechanical properties of the printed samples was then examined in unconfined compression stress-strain tests of 1 × 1 × 1 cm^3^ sized samples. To evaluate the adhesion of the material between printed layers, compression measurements were performed along the parallel and perpendicular directions to the printing lines.

**Results:**

At a laser density of 70 mW/cm^2^, Young’s modulus was approximately 6 MPa up to a maximum compression of 20% in the elastic regime for both the parallel and perpendicular measurements. These values were within the range of biological cartilage values. Cytotoxicity tests performed with Vero cells confirmed the cytocompatibility.

**Conclusions:**

We printed a partial tracheal model using optimized printing conditions and proved that the materials and methods developed may be useful for printing of organ models to support surgery or even to produce customized tracheal implants, after further optimization.

**Electronic supplementary material:**

The online version of this article (10.1186/s41205-019-0049-8) contains supplementary material, which is available to authorized users.

## Background

There are several potential health-related applications of 3D printing [1], most of which are in the field of neurosurgery [2], orthopaedics [3], spinal surgery [4], maxillofacial surgery [5], tissue engineering [6], indirect fabrication of medical devices [6], cell seeding and culturing [7], cardiac surgery [8] and cranial surgery [9, 10], where 3D printing can be used to print the final implant. 3D printing can also be used as an aid in 3D models to help visualize complex medical cases, in addition to assisting student teaching and patient education also allows health professionals to practice certain procedures [1], which can be complemented by the fabrication of anatomical models for pre-surgical planning [6]. 3D printing of customized prosthetics to replace damaged regions of bones, organs, cartilage or tissue is in high demand to enable prosthesis integration. However, resolution of 3D printing technologies is not a limiting factor, there is a need for new biocompatible materials that can fulfil the required specificities of different applications, such as cartilage [1, 6, 11].

Hydrogels are cross-linked networks of hydrophilic polymers, which are capable of absorbing water up to thousands of times their dry weight [12]. They are also biocompatible and can be delivered into the body through minimally invasive methods [12]. Although hydrogels are the most extensively studied materials for cartilage replacement, implants with properties that mimic natural cartilage are some way off. Several ways to obtain hydrogels in a 3D form have been reported [11]. Commonly used methods include dispensing two reactive components using mixing nozzles, inducing cross-linkage through heat or UV light [1, 6, 11] or delivering one material to a plotting reactive medium to finish the curing reaction. Hydrogels can be fabricated in various ways, such as radiation, freeze-thawing, chemical cross-linking or thermal annealing [13]. Some examples of biocompatible hydrogels are polyethylene glycol diacrylate (PEGDA), which is a blank slate hydrogel that jellifies rapidly at room temperature in the presence of a photoinitiator and UV light. Since PEGDA gels are hydrophilic and elastic, and can be mixed with a variety of biological molecules, they constitute powerful tools for bioprinting. PEGDA gels are also biologically inert, and their mechanical properties can be adjusted over a large range of Young’s moduli. Poly (vinyl alcohol) (PVA) and alginate are other hydrogels widely used in biomedical applications [14, 15]. The mechanical properties of PVA can be enhanced by cross-linking with glutaraldehyde [14]. Alginate becomes a hydrogel when an aqueous alginate solution is mixed with divalent cations due to ionic cross-linking [15]. The blocks of guluronate in alginate chains bind to the divalent cations, and a gel structure forms by the junction of functional groups from separated polymer chains [15]. The cross-linking of hydrogels is essential to provide stability, flexibility and support the required strength of applications. That is possible when established bonds and networks are resilient to the breakage of covalent bonds.

Composite hydrogels obtained by chemical cross-linkage of polyethylene glycol (PEG) and physical cross-linkage of polyvinyl alcohol (PVA) have been investigated previously [16]. As compared with pure PVA hydrogels, which have tensile strength of only 0.17 MPa at ultimate elongation of 312%, the incorporation of chemically cross-linked PEG improves the tensile strength with the increasing PEG content [16]. There has been little research on 3D printing by extrusion of pastes combined with in situ UV laser light cross-linking or the use of riboflavin-triethanolamine (TEOHA) mixtures as photoinitiators [17]. Nguyen et al. [17] recently demonstrated the polymerization of a mixture containing PEGDA (as the polymer), riboflavin (as the photoinitiator) and TEOHA (as the co-initiator) using two-photon polymerization and a stereolithography printing technique. Pre- or post-printing cross-linking has been attempted previously. The former consists of focusing UV light on a container, usually a syringe, whereas the latter involves focusing UV light on already printed materials. However, UV light through the extrusion region requires a transparent nozzle/needle and a cross-linking variant in situ [18].

In this work, we studied the 3D extrusion of pastes containing PEGDA, sodium alginate (SA) and a photoinitiator (B2VT) consisting of a solution of riboflavin and TEOHA using a UV laser coupled to a syringe that contained the polymer mixture to be printed. This work focused on the influence of laser power on the mechanical properties of the printed samples and its optimization. In the approach applied, after each hydrogel printed layer, the UV laser scanned the printed region at the same speed as the paste extrusion. A 3D model of a trachea was printed under optimized conditions, including a segment of a life-sized trachea. This work demonstrates that it is possible to print models that can be used as an aid to surgery and to print customized implants after further improvements and studies.

## Materials and methods

### Ink preparation

Ink gels for 3D printing were prepared by mixing 10.8 mg of calcium sulphate (CaSO_4_), 1 ml of ultrapure water and 2.5 ml of 34.78% wt/v PEGDA (Mn = 575, Sigma-Aldrich) solution in ultrapure water, 2.5 ml of 5% v/v SA (90.8%, Biochemica) solution in ultrapure water and 1 ml of B2VT solution. CaSO_4_ was weighed before the addition of 1 ml of ultrapure water and 2.5 ml of the PEGDA solution under constant stirring. Subsequently, while maintaining stirring, 2.5 ml of the SA solution were added to enhance cross-linkage of the blend. Finally, the B2VT solution was added to absorb UV radiation, and the mixture was loaded into a 20 cc syringe and left to rest in an upright position for up to 12 h until the liquid became gel like. The B2VT solution (10 ml) was prepared as follows: 9.5 mg of riboflavin (98%, Sigma-Aldrich), 3.1 g of TEOHA (98%, Sigma-Aldrich) and 10 ml of ultrapure water. The materials were placed in separate containers and weighed. Ultrapure water (5 ml) was then added to each container under magnetic stirring. Both solutions were kept at room temperature. After stirring for 30 min, the two solutions were mixed together and stirred for another 30 min.

### Printing procedure

**Fig. 1:**
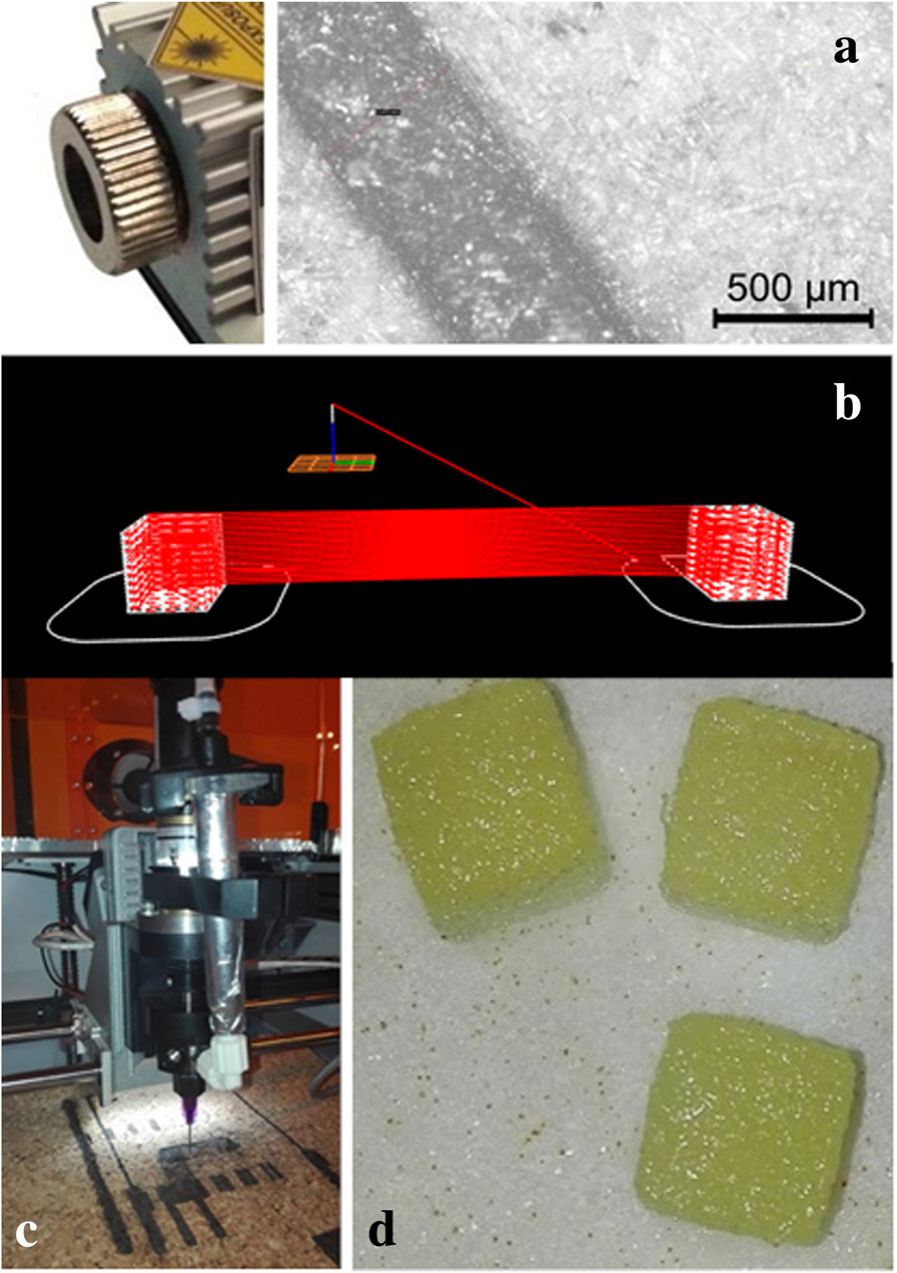
**a** Photo of the UV laser used and the scribing width of groove made in a wood piece with the maximum power: **b** file (.gcode) for printing and UV laser scanning: **c** photograph of printer; **d** and example of printed samples for compression tests (1x1x1 cm^3^)

A home-made 3D plotter was built [19] by integrating a syringe controlled by compressed air and a UV laser (3.8 W laser head, JTech Photonics) of variable power up to a maximum of 3.8 W. The printing process was performed sequentially, with a printed paste layer followed by laser scanning at the same speed as the printing paste extrusion (15 mm/s) and the same line width (0.3 mm), with intensities in the range of 0.4 to 2.0 W. A 20 cc syringe with a 0.3 mm needle was used with a pressure of 1.8 bar. The samples were designed using Blender and Cura 2.4.0, which created a G-code file that was later read by the printer. In this file, the laser power was determined by a number between 0 and 255, which corresponded to a power range between 0 and 3.8 W. The laser emission wavelengths were in the range of 435 to 455 nm, which included one UV-Vis absorption peak of PEGDA/B2VT [20]. Figure 1 shows an image of the laser used and an example of the laser scribing a piece of wood at the maximum power of 3.8 W (Fig. 1a). Figure 1b shows the movement of the syringe and that of the laser in parallel during the printing process, and Fig. 1c shows the syringe used. Examples of the samples produced for mechanical tests are shown in Fig. 1d. The selected extrusion air pressure and printing parameters shown were optimized previously to obtain denser printing for the blend used according to the size of the syringe and characteristics of the step motors of the printer. The mechanical compression as a function of the laser power were examined while maintaining all other printing parameters at optimized settings.

**Fig. 2:**
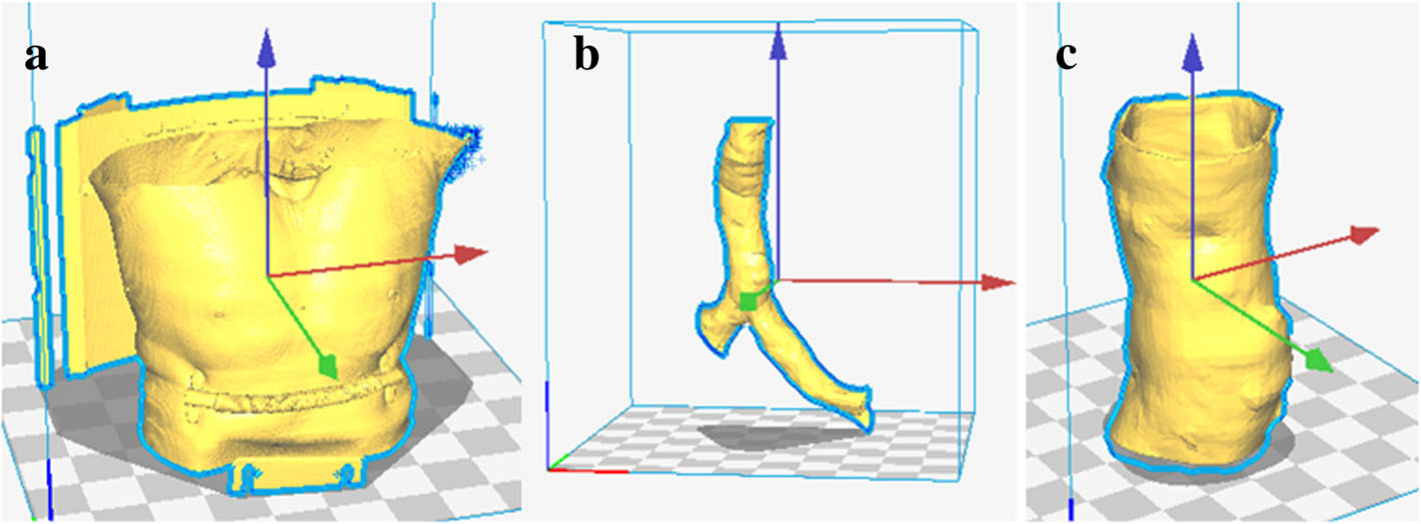
3D models in .stl format based on the patient’s CT scan: **a** initial model received; **b** model after some modifications; **c** model ready to print

To print a 3D tracheal model, data from an undisclosed patient’s computed tomography (CT) scan were used. The information retrieved from the scan was rebuilt through the files in DICOM® (Digital Imaging and Communications in Medicine) format and saved in.stl format, used for 3D printing, and converted into G-code, the language that gives all the instructions to 3D printer. This final file was saved in a .gcode format using Cura 2.4.0. The .gcode file was then altered using software developed to incorporate a UV laser scan between each printed layer. This enabled each layer to be reticulated and vulcanized to the layer beneath. Examples of the .gcode images created are shown in Fig. 2, with different views (top, front and top). The y-axis of the laser was offset by − 70 mm to ensure it was in focus. The .gcode files were saved to a memory card, which was then placed in the printer’s computer. The objects were printed by extrusion and left to dry until completely solid.

### Mechanical tests

Unconfined compression tests were performed on printed dried samples (1 × 1 × 1 cm^3^) using a Shimadzu AG-50 kNG mechanical testing machine at 0.5 mm/min. The material was placed between two parallel steel plates. Perpendicular (⊥) and parallel (//) tests were then conducted in which a uniaxial force was applied perpendicular or parallel to the printed layers. At each laser power, a set of between five and seven samples were tested. A section of each sample was measured before and after the tests using a digital calliper. The load was applied to the samples until the strength started to decrease. The compressive force versus the sample height change was taken as representing the true stress/strain value in accordance with previous research [21].

### Cytotoxicity tests

Cytotoxicity studies were performed using African green monkey kidney epithelial cells, known as Vero cells. The cells were cultured in Dulbecco’s Modified Eagle’s Medium (catalogue # D5030 Sigma-Aldrich), supplemented with GlutaMAX (#35050–038), 10% v/v fetal bovine serum (#10270106), 100 units/mL of penicillin and 100 μg/mL of streptomycin (#15140122), all from Life Technologies. Then, 12 k cells were seeded per well in a 96-well plate. All procedures were performed inside a biological safety cabinet (ESCO Labculture class II). Cultures were incubated at 37 °C in a 5% carbon dioxide humidified atmosphere incubator (SANYO MCO-19AIC (UV)).

In the cytotoxicity assay, the extract method was used according to International Standard ISO 10993-5. The tested samples were weighed, sterilized by immersion in an aqueous solution of ethanol 70% v/v, left to dry and irradiated under UV light (254 nm) for 30 min. Each sample was placed in a sterilized tube to which culture medium was added in a proportion of 10 mg/mL. The samples were kept immersed at 37 °C for 24 h. The cell culture extracts were removed and used to replace the medium in the wells containing seeded 24 h earlier. Negative (viable cells) and positive (cells in a cytotoxic environment) controls were established by culturing cells with culture medium and culture medium with 20% DMSO, respectively. Five replicas of each condition were used. The cells were then incubated with the extracts for 24 h, after which a colorimetric viability assay was performed. The media in the culture wells were replaced by culture medium with 10% resazurin (Alfa Aesar) solution (0.2 mg/mL in phosphate buffered saline), and the cells were incubated for 3 h. Resazurin, a blue dye (λabs = 601 nm), was reduced by dehydrogenase enzymes in metabolically active cells, given rise to resorufin, which had a pink colour (λabs = 571 nm). The absorbance measured at 570 nm, using a reference wavelength of 600 nm (Biotex ELX 800UV microplate reader), corrected by the medium control, was considered proportional to cell viability. The relative viability under the tested conditions was deduced by the ratio of the absorbance measured for that condition and the absorbance of the negative control. The combined standard uncertainty was calculated by propagation of uncertainties.

## Results

**Fig. 3:**
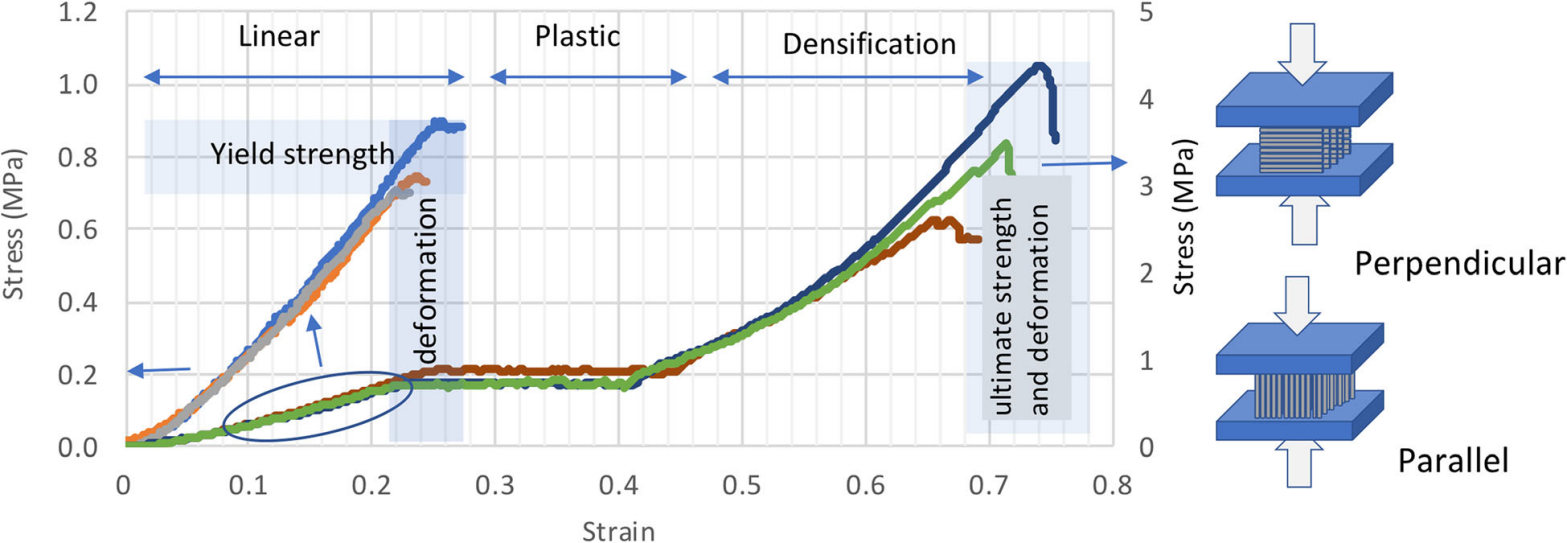
Stress-strain curves for unconfined compression tests of cubes reticulated at 941 mW of perpendicular samples – first slope for each curve on the left axis and complete curve for each sample on the right axis. In blue are highlighted the regions where yield strength, deformation and ultimate strength and deformation were obtained while arrows represent the linear, plastic and densification regions of the curves. Beside the figure is sketched the applied force for perpendicular and parallel measurements in respect to printing lines

The stress-strain curves obtained from the compression tests performed perpendicularly to the printing plane, using two parallel flat contact surfaces and cubic samples (1 × 1 × 1 cm^3^) with planar side walls are illustrated in Fig. 3. For simplicity, only three samples are shown, as these represent the curves obtained for the other samples and those obtained for the parallel measurements. The stress values in the linear region of the curve, where the yield strength was measured, are represented on the left axis, whereas the right axis corresponds to the stress values for the entire curve.

In the case of additive manufacturing, the 3D sample was obtained by stacking layers over layers of material. Thus, adhesion between the layers constrained the mechanical properties. In the fabrication process used herein, the printing conditions were set to obtain maximum density with the selected blend, and cross-linking was obtained using a UV laser that scanned the printed layer just after extrusion, while following the same trajectory of the extruder nozzle. The gel printing followed by the UV laser scanning process was first verified by printing one, two or more layers to determine whether the hydrogel maintained three dimensions; the colour changed from transparent to white, thereby indicating that cross-linkage occurred; and the hydrogel could be removed from the substrate and handled without breaking (Additional file 1: Figure S1).

Up to around 20–25% strain, the stress (σ) versus strain (ɛ) curve exhibited a linear region, from which the elastic modulus or Young’s modulus was determined from the slope. The yield strength, respective deformation values, and maximum strength and corresponding deformation before collapse were also determined [17, 22]. The plastic deformation plateau followed the yield behaviour, in common with foams and most polymers [22]. Upon a further increase in the compression force, the material underwent densification, corresponding to a second slope in the stress versus strain curve, culminating in failure of the connection between the polymer chains at ultimate strength. The yield deformation decreased, and deformation was more pronounced when the compression force was applied perpendicularly to the plane of the printed layers (Additional file 1: Table S1).

**Fig. 4:**
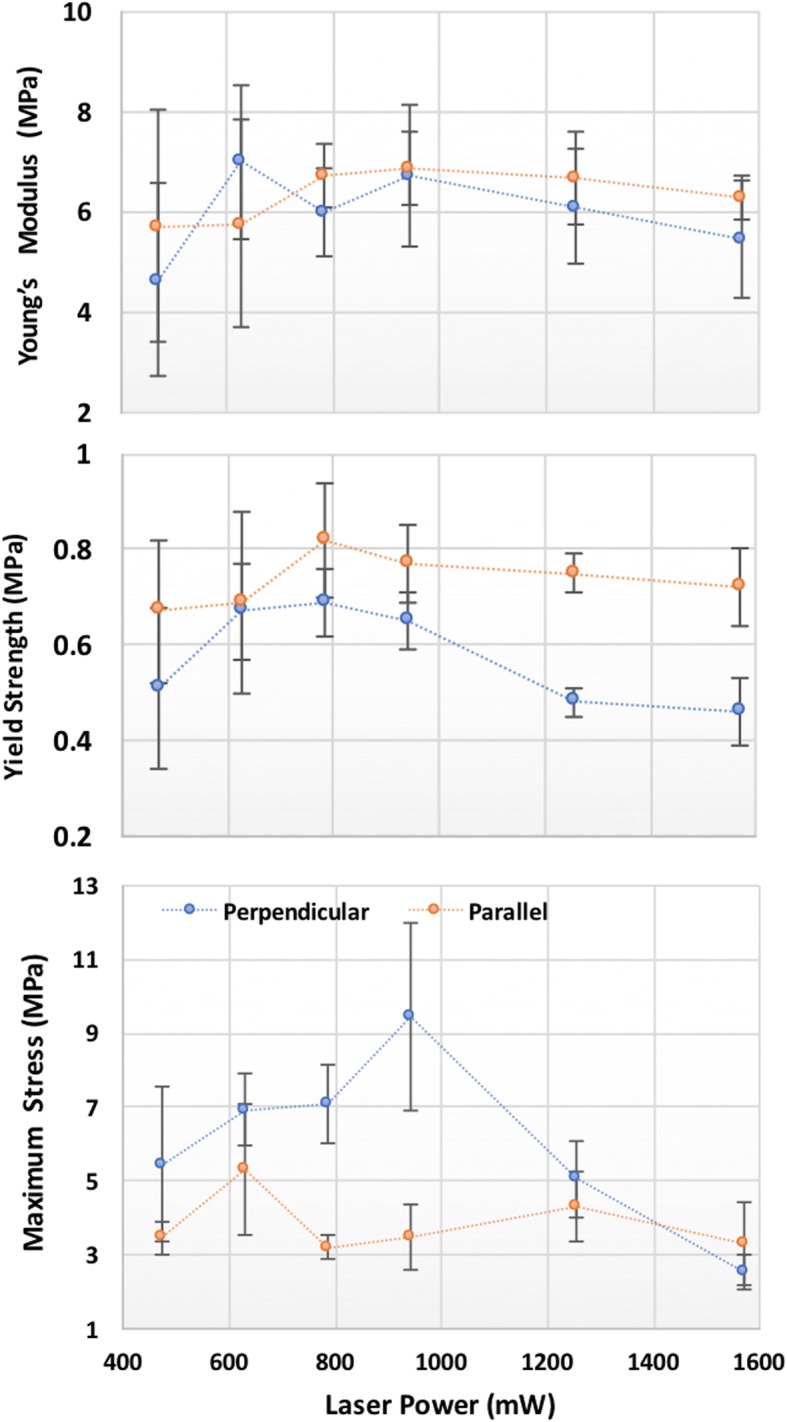
Yield strength, Young’s modulus and maximum strength for parallel and perpendicular compression tests as a function of UV laser power. The error bar represents the measurements standard deviation (STD)

**Fig. 5:**
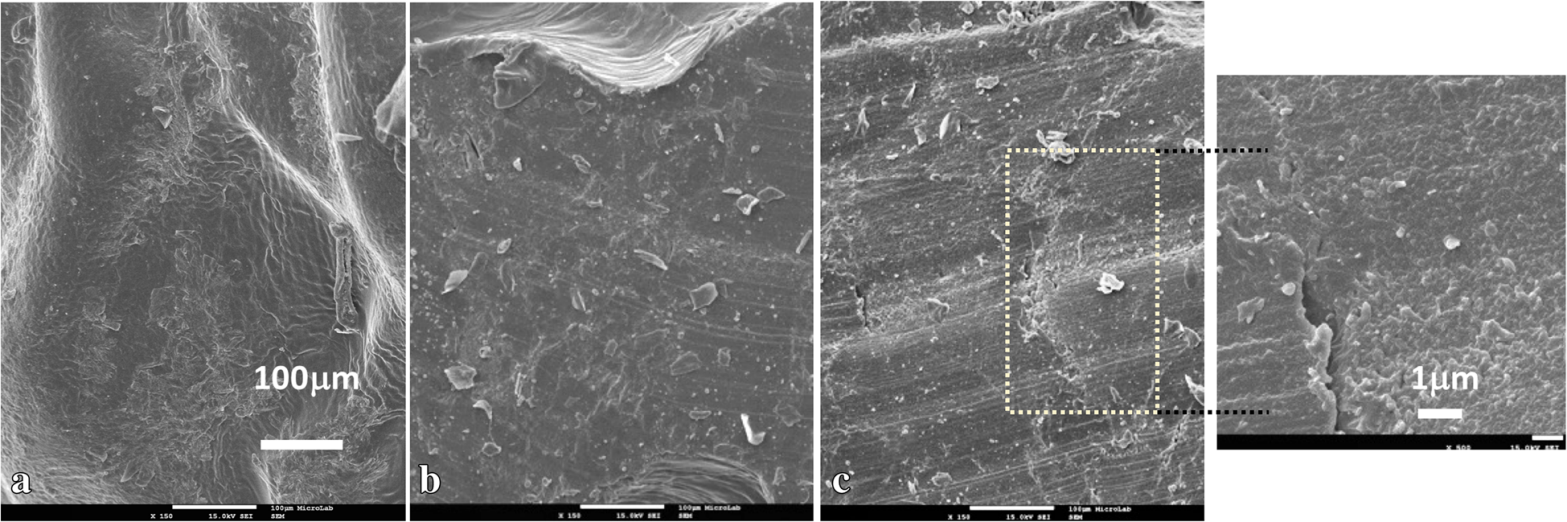
SEM images of samples produced with UV laser power of: 0.9 W- **a** top view and **b** cross section; 1.6 W - **c** cross section

The average mechanical parameters (i.e. Young’s modulus, yield strength and ultimate compressive strength) obtained from the measurements of the five different samples in the parallel and perpendicular compression tests as a function of the laser power are shown in Fig. 4. A top view and cross-section SEM image of the 0.9 W samples are shown in Fig. 5a and b, respectively. The effect of increasing the laser intensity is shown in Fig. 5c, where a failure region perpendicular to the printing lines can be seen.

**Fig. 6:**
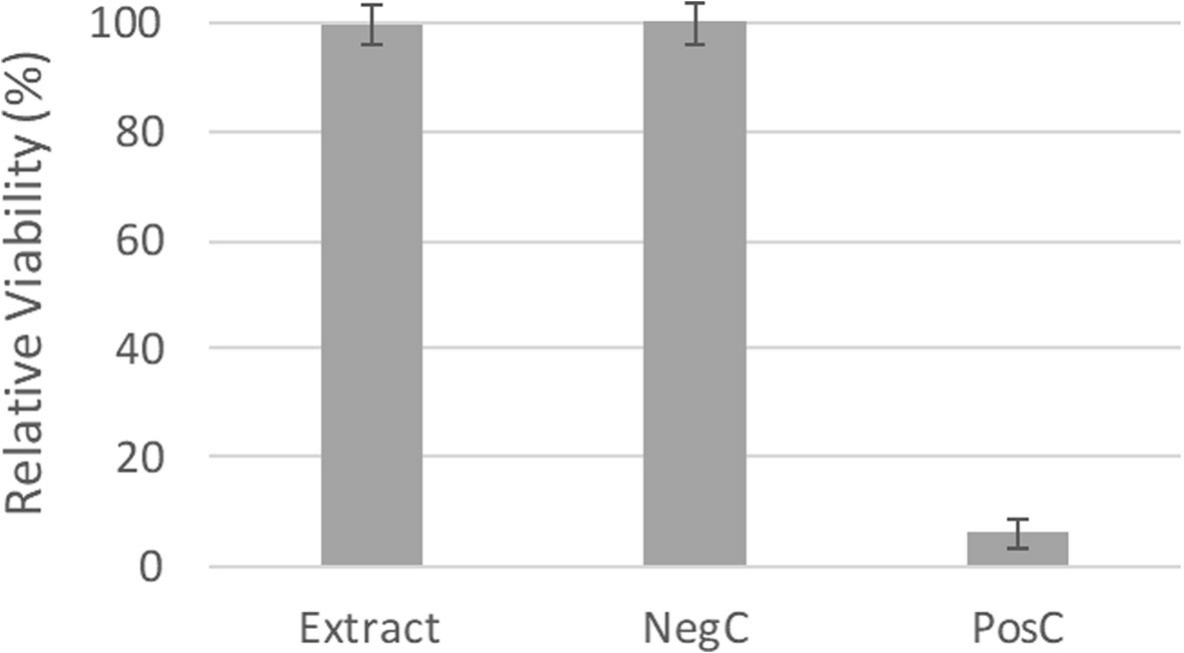
Results of the cytotoxicity assay: relative viability of *Vero* cells incubated with extract, culture medium (NegC) and culture medium with 20% DMSO (PosC)

Cell viability was used as an indicator of cytotoxicity and assessed using the resazurin reduction test. Figure 6 shows the relative viability of cells exposed to an extract of a sample cross-linked at laser power of 800 mW. The results obtained for the extracts of samples produced under other laser powers were similar to those obtained at 800 mW. The cell viabilities obtained in the negative control (viable cells) and positive control (cells exposed to a cytotoxic environment comprising culture medium with 20% DMSO) are displayed in Fig. 6. Based on the findings, we conclude that the viability of cells incubated with the extracts and the negative control was the same. This result points to the absence of leached cytotoxic compounds from the samples.

**Fig. 7:**
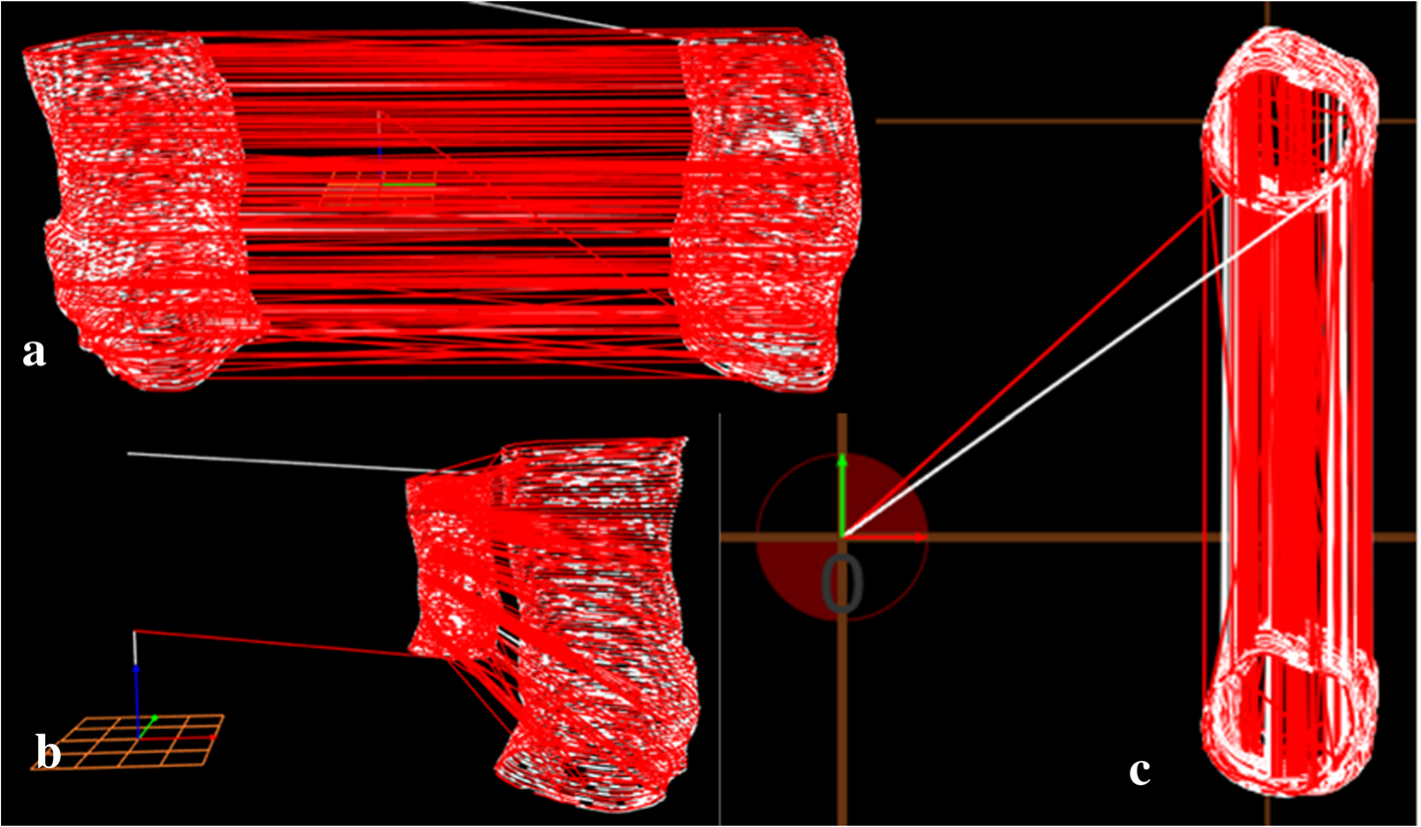
3D models in .gcode format with intercalated laser layers: **a** view from the right; **b** front view; **c** top view

**Fig. 8:**
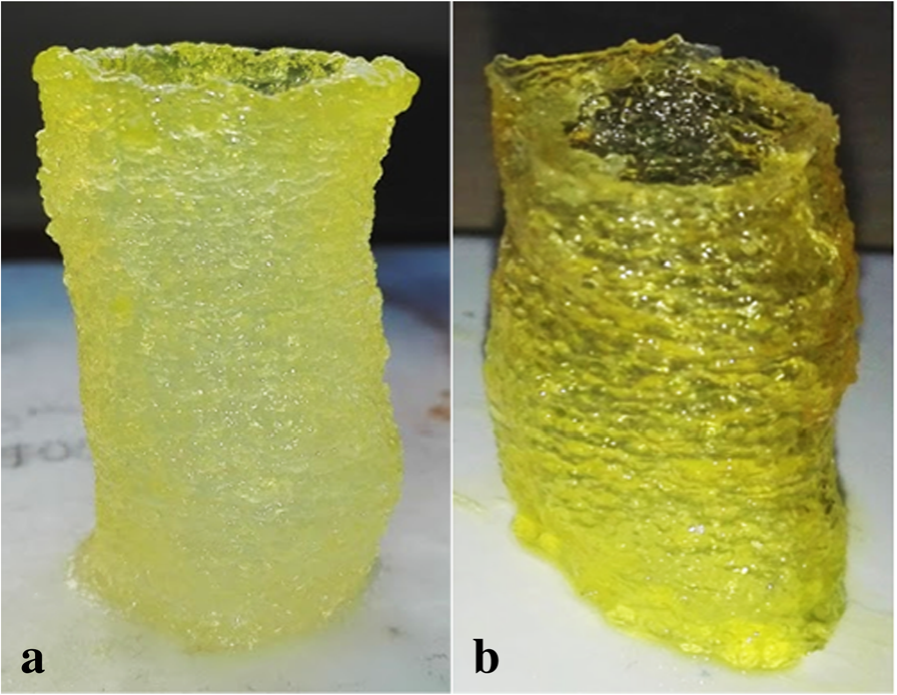
Tracheal section 3D printed with simultaneous UV reticulation: **a** right after printing (height = 38.5 mm, width = 19.24 mm, thickness = 0.5 mm) and **b** after 72 h

The original files of the patient’s trachea model are shown in Fig. 2. The file was modified in Blender, software used for creating 3D models (Fig. 7). The 3D printing of a tracheal segment is shown in Fig. 8.

## Discussion

In the present work, there were only small differences between the stiffness values of printed samples determined in both the perpendicular and parallel compression tests. Thus, Young’s modulus can be considered to be almost isotropic and independent of the laser power, suggesting that a homogeneous polymer mixture and cross-linking were achieved at laser power in the range of 400–1600 mW. Outside this range (below 400 mW), it was impossible to obtain a solid object, and cross-linking of PEGDA was incomplete. Consequently, the gel spread when the layers were superposed.

Laser power above 1700 mW led to brittle 3D samples. High laser energy caused point defects or glass transition of the polymers, which resulted in fragile regions in the samples at the site of failure, leading to a marked decrease in yield strength. Laser power in the range of 600–1000 mW was optimum for maximizing the mechanical properties and cross-linking of the layers. Uniform and isotropic E values of 6–7 MPa and yield strength of 0.7–0.8 MPa were obtained within this power laser range. Above this laser power, the maximum strength decreased on average, and the difference between the parallel and perpendicular values increased. The linear deformation reached 20% at laser power up to 900 mW but decreased to 15% or lower at higher laser powers. Contrary to the stiffness values, the ultimate strength showed anisotropy between the parallel and perpendicular measurements. When stress was applied parallel to the layers, the weak region of the samples was the layers’ junction. Thus, when the deformation force was perpendicular to the layers, layer detachment can occur, and the maximum power is affect by the interpenetration and cross-linkage between the printed lines. When these were sufficiently strong, isotropy was solely dependent upon the porosity of the material. However, the variation of perpendicular and parallel strength in the range of 600–1000 mW was smaller than that observed at a laser power output above 1600 mW. The optimized laser power of 600–1000 mW corresponded to a power density of 42–70 mW/cm^2^.

In this study, we focused on the mechanical properties of PEGDA, SA and a B2VT mixture with a fixed composition the one that have shown the better gelation properties when extruded by a syringe. Bashir et al. [23] reported a decrease in the elastic modulus from 500 kPa to 5 kPa when the molecular weight of PEG increased from 0.7 kDa to 10 kDa in PEGDA hydrogels printed using a stereolithography technique. Rennerfeldt et al. [21] studied the influence of different percentages of PEGDA (10, 20 and 30%) and three molecular weights (2 kDa, 3.4 kDa and 6 kDa) in mechanical compression tests of mould-produced samples containing 0, 2 and 5% agarose. Samples of 0% agarose showed maximum stress of 3 MPa with 20% PEGDA and a molecular weight of 6 kDa, and the variation in maximum strain was almost independent of the percentage of PEGDA, with increases from 0.6 to 1 when the molecular weight increased from 2 kDa to 6 kDa. Duan et al. [24] described a 3D printed alginate/gelatin hydrogel in a 3D grid of lines about 1 to 2 mm apart. In their study, the ultimate tensile strength of the hydrogel decreased from 0.84 MPa to 0.12 MPa strength, and the elastic modulus decreased from 1.44 to 0.99 MPa after 24 h of production but remain unchanged for 7 days. Yasar and Inceoglu [25] studied mechanical compression properties of PEGDA rods made by moulds and UV photopolymerization. In their study, Young’s modulus increased from 3.1 to 57 MPa, and maximum strength increased from 0.5 to 10 MPa in accordance with changes in the percentages of PEGDA in water from 20 to 100%. In a recent study on the mechanical strength of alginate/poly (acrylamide-co-acrylic acid)/Fe^3+^(SA/P (AAm-co-AAc/Fe^3+^), the authors reported tensile strength and strain values of 3.24 MPa and 1228%, respectively [26]. Eu-containing poly (vinyl acetate) and PVA triple physical cross-linked hydrogels exhibited 2 MPa of compressive stress [27]. GelMA hydrogels with a compressive modulus of 288.24 ± 62.34 kPa and Young’s modulus of 264.74 ± 11.08 kPa at 25 °C have also been reported [28]. A wood hydrogel with 65 wt% of water weight content showed significantly improved fracture tensile strength and Young’s moduli of 36 and 310 MPa in the plane of the wood fibres, and 0.54 and 0.135 MPa in the perpendicular plane to the fibres [29].

State of the art hydrogels made by 3D printing techniques [30] clearly show that their mechanical properties are enhanced by cross-linking, which can be achieved by various methods, such as temperature, chemical reactions or photopolymerization. Cross-linking using photopolymerization requires the use of a photoinitiator. The main advantages of this method are the rapid formation of a hydrogel under room temperature and tuning of the cross-linking reactions by the light exposition time and intensity [13, 31]. Furthermore, only the radiated areas are cross-linked, which allows the construction of hydrogels with complex geometries and structures [13, 31]. The double bonds of unsaturated groups of some compounds, such as (meth) acrylates, are highly reactive, and excitation with light promotes radical chain-growth polymerization. Examples are reactions of hydroxyl, carboxyl and amino groups of water soluble polymers with acryloyl chloride, glycidymethacrylate forming vinlyl groups [32]. For biomedical applications, the photoinitiator must be cytocompatible. The photoinitiators Irgacure [33], riboflavin [17] and eosin [34] absorb radiation in the UV range of 250–370 nm and visible range of 405–550 nm. As UV radiation exposure is considered dangerous to DNA [35], a photoinitiator absorbing in the visible range is preferable for cross-linking of hydrogels containing cells. Thus, we used B2T2 in our experiments and added CaSO_4_ to in the gel mixture in an attempt to increase the efficiency of cross-linkage of PEGDA. Park et al. [36] reported a beneficial effect of 0.1 M CaCl_2_ and Na_2_HPO_4_ on the reduction of temperature and gelation time of a methycellulose hydrogel. A recent research work also showed that divalent cations, such as Ca^2+^, bound to guluronate blocks of polymer chains and facilitate the junction of guluronate blocks of adjacent polymer chains. Several attempts to enhance the mechanical properties of hydrogels have been reported in the literature [37–41]. Some examples include reinforcement of alginate; gelatin hydrogels reinforced with bioglass [37] and hydroxyapatite [38]; alginate reinforced with biphasic calcium phosphate [39]; PEGDA reinforced with hydroxyapatite [40]; and PEGDA, alginate and gelatin reinforced with laponite [41].

Previous research reported that the mechanical properties of PEGDA hydrogels that mimicked cartilage were highly dependent on the fabrication process. Studies also showed that various factors, such as the UV exposure time, intensity and photoinitiator concentration, influenced PEGDA cross-linking and that the porosity of the film depended on the fabrication method. The surface morphology of our 3D printed samples was dense. The latter may explain the good mechanical properties of the samples. At a higher laser intensity, a failure region occurred perpendicular to the printing lines (Fig. 5c). As the compressive effort was applied along the same direction, the corresponding shear stresses generated along the print lines must have been responsible for the fracture. Thus, failure of the printed part was due to cohesive failure within each individual layer and not to adhesive failure between the different layers (layer detachment).

In the present work, the mechanical properties obtained in the compression stress tests (Young’s modulus of 6–8 MPa and maximum strength of 7–11 MPa) were above the range values of biological cartilage (1.9 MPa Young’s modulus and maximum strength of 3 MPa) [42]. In future work, we will investigate the influence of variations in porosity on the mechanical strength, together with fabrication parameters. In this study, all the parameters were fixed, except the laser power, and we focused only on the mechanical strength of the printed samples. Future studies will correlate the laser power with sample swelling, as the toughness of samples in contact with body fluids is very important for cartilage applications.

In the present work, several tracheal samples were printed with different wall thicknesses. As the wall thickness changed from 0.5 mm to 1.5 mm, the compliance of the trachea was reduced, and it became less fragile.

## Conclusions

In this study, a tracheal prosthesis was 3D printed using a UV laser cross-linking method and a PEGDA, SA and B2VT mixture. The laser power intensity was in the range of 40–70 mW/cm^2^, and the scan speed was 15 mm/s. This resulted in optimisation of Young’s modulus of around 6–7 MPa, yield strength of 0.7–0.8 MPa and maximum strength of 7–11 MPa, which corresponded to yield deformation of 20% and 70% deformation before failure. We believe that both the polymer mixture and printing process described in this study are promising methods for creating personalized cartilage implants in the future.

## Additional file

Additional file 1: Complementary information on UV cross linkage and mechanical properties. (ZIP 464 kb)

## Data Availability

The datasets used and/or analysed in the current study are available from the corresponding author on request.

## Abbreviations

CaSO_4_: Calcium sulphate
CT: Computed tomography
DMSO: Dimethyl sulfoxide
PEG: Polyethylene glycol
PEGDA: Polyethylene glycol diacrylate
PVA: Poly (vinyl alcohol)
RF: Riboflavin
SA: Sodium alginate
TEOHA: Triethanolamine
