# Supplementary material for: Customized tracheal design using 3D printing of a polymer hydrogel: influence of UV laser cross-linking on mechanical properties
Source: 3D Print Med. 2019 Aug 2;5:12. doi: 10.1186/s41205-019-0049-8 (PMC6743139; doi:10.1186/s41205-019-0049-8)
Supplement: Supplementary file 1 — Complementary information on UV cross linkage and mechanical properties. (ZIP 464 kb) [file 41205_2019_49_MOESM1_ESM.zip › Additional file Table S1.docx]

**Supplementary information**

First tests with the 3D plotter were performed and as the UV laser effect on the reticulation of polymer mixture B was unknown, preliminary trials with different number of printed layers were done. Control pieces were printed with 1, 2, 3 and 4 layers to determine the effect of UV reticulation and test the material’s printing - Figure S1. Further improvements were tested by refining the ratio between components as seen in Table S1.


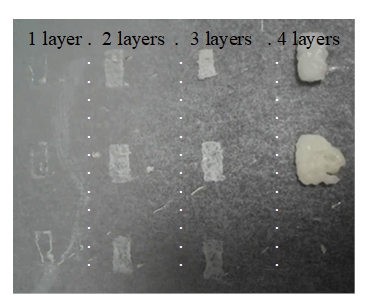


**Figure S1** – 3D printed and UV cross-linked samples printed with 1, 2, 3 and 4 layers

**Table S1** – Mechanical properties as a function of Laser power

| **Laser Power**  **(mW)** | **Meas. Direct.** | **Max Stress (MPa)** | **Max**  **Strain**  **(%)** | **Max**  **Force**  **(Kg)** | **Densification slope**  **(MPa)** | **Young’s Modulus**  **(MPa)** | **Maximum Elastic strain (%)** | **Yield Strength**  **(MPa)** |
| --- | --- | --- | --- | --- | --- | --- | --- | --- |
| 470 | \|\| | 3.5 ± 0.5 | 74.0 ± 3.0 | 11.4 ± 1.3 | 39.9 ± 8.7 | 5.7 ± 2.3 | 20.0 ± 0.8 | 0.7 ± 0.2 |
|  | ⊥ | 5.5 ± 2.1 | 78.6 ± 2.5 | 26.3±10.4 | 106.7 ± 37.9 | 4.6 ± 1.9 | 24.7 ± 9.6 | 0.5 ± 0.2 |
| 627 | \|\| | 5.3 ± 1.8 | 69.0 ± 3.9 | 17.1 ± 3.3 | 81.5 ± 39.7 | 5.8 ± 2.1 | 17.2 ± 2.7 | 0.7 ± 0.2 |
|  | ⊥ | 6.9 ± 1.0 | 79.4 ± 0.7 | 27.1 ± 5.5 | 101.9 ± 21.4 | 7.0 ± 1.5 | 19.2 ± 3.1 | 0.7 ± 0.1 |
| 784 | \|\| | 3.2 ± 0.3 | 64.4 ± 2.6 | 9.9 ± 1.5 | 37.4 ± 6.6 | 6.7 ± 0.6 | 20.5 ± 2.2 | 0.8 ± 0.1 |
|  | ⊥ | 7.1 ± 1.1 | 74.6 ± 5.6 | 24.2 ± 2.7 | 115.6 ± 23.6 | 6.0 ± 0.9 | 19.4 ± 3.4 | 0.7 ± 0.1 |
| 941 | \|\| | 3.5 ± 0.9 | 70.3 ± 3.6 | 11.3 ± 3.6 | 40.5 ± 12.2 | 6.9 ± 0.7 | 23.4 ± 1.4 | 0.8 ± 0.1 |
|  | ⊥ | 9.5 ± 2.5 | 66.9 ± 6.1 | 35.2 ± 8.1 | 177.9 ± 62.4 | 6.7 ± 1.4 | 15.0 ± 3.6 | 0.7 ± 0.1 |
| 1255 | \|\| | 4.3 ± 1.0 | 67.1 ± 3.5 | 13.0 ± 3.2 | 46.2 ± 13.1 | 6.7 ± 0.9 | 16.0 ± 2.3 | 0.8 ± 0.01 |
|  | ⊥ | 5.1 ± 1.0 | 70.3 ± 6.3 | 23.8 ± 6.7 | 74.1 ± 23.6 | 6.1 ± 1.1 | 13.4 ± 1.3 | 0.5 ± 0.01 |
| 1569 | \|\| | 3.3 ± 1.1 | 65.6 ± 2.8 | 11.7 ± 4.9 | 45.5 ± 17.0 | 6.3 ± 0.4 | 18.7 ± 4.6 | 0.7 ± 0.1 |
|  | ⊥ | 2.5 ± 0.5 | 50.6 ± 2.0 | 14.4 ± 1.8 | 28.4 ± 4.8 | 5.5 ± 1.2 | 14.1 ± 1.7 | 0.5 ± 0.1 |
