# Supplementary figures and images for: Customized tracheal design using 3D printing of a polymer hydrogel: influence of UV laser cross-linking on mechanical properties
Source: 3D Print Med. 2019 Aug 2;5:12. doi: 10.1186/s41205-019-0049-8 (PMC6743139; doi:10.1186/s41205-019-0049-8)

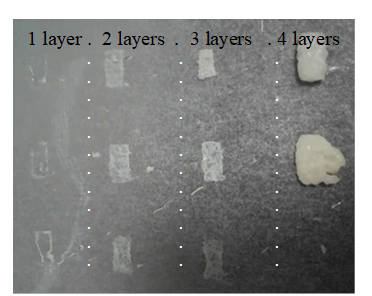

Supplement: Supplementary file 1 — Complementary information on UV cross linkage and mechanical properties. (ZIP 464 kb) [file 41205_2019_49_MOESM1_ESM.zip › Figure SI.tif]
